# Healthy worker hire and survivor effects in a cohort of medical radiation workers

**DOI:** 10.1093/ije/dyae130

**Published:** 2024-10-04

**Authors:** Won Jin Lee, Jaeho Jeong, Ye Jin Bang, Young Min Kim

**Affiliations:** Department of Preventive Medicine, Korea University College of Medicine, Seoul, South Korea; Department of Statistics, Kyungpook National University, Daegu, South Korea; Department of Preventive Medicine, Korea University College of Medicine, Seoul, South Korea; Department of Statistics, Kyungpook National University, Daegu, South Korea

**Keywords:** Bias, cohort, hospital workers, ionizing radiation, occupational exposure

## Abstract

**Background:**

The healthy worker effect may distort the association between exposure and health effects in workers. However, few studies have investigated both the healthy worker hire and survival effects simultaneously, and they are limited to mortality studies in male workers.

**Methods:**

We utilized a data set comprising South Korean diagnostic medical radiation workers registered in the National Dose Registry between 1996 and 2011, and merged it with mortality and cancer incidence data. Standardized mortality ratios (SMRs) and standardized incidence ratios (SIRs) were computed for comparison with the general population. To account for time-varying confounders influenced by prior occupational radiation exposure, we applied g-estimation using structural nested accelerated failure time models and compared the outcomes with those from Weibull regression.

**Results:**

A total of 1831 deaths and 3759 first primary cancer cases were identified among 93 918 workers. Both male (SMR = 0.44; 95% CI: 0.42, 0.46) and female workers (SMR = 0.53; 95% CI: 0.46, 0.60) showed lower mortality rates compared with national rates. In the SIR analysis, male workers exhibited reduced risks of solid cancer whereas female workers had increased risks. The g-estimation-derived hazard ratios (HRs) from radiation exposure exceeded those from Weibull regression estimates for all-cause death (HR = 2.55; 95% CI: 1.97, 3.23) and all-cancer incidence (HR = 1.96; 95% CI: 1.52, 2.55) in male workers whereas female workers showed the opposite results.

**Conclusions:**

Comprehensive consideration of the healthy worker effect by sex is essential for estimating the unbiased impact of occupational exposure on health outcomes, notably in studies focusing on male mortality.

Key Messages

- This study was aimed to investigate the healthy worker effect to provide deeper insights into the exposure–response relationship in epidemiological research.
- Substantial healthy worker hire and survivor effects were observed in both male and female radiation workers with different patterns.
- The healthy worker hire and survivor effects were larger on mortality than incidence indexes.
- Neglecting the healthy worker effect may introduce bias in estimating the exposure–response associations, particularly in male mortality studies.

## Introduction

The healthy worker effect poses a potential challenge in occupational cohort studies by distorting the association between exposure and health outcomes in workers.^1^^,^^2^ The healthy worker hire effect, selecting relatively healthier individuals during hiring, can be addressed by internal comparisons within the working population. However, the healthy worker survivor effect, ongoing selection of workers out of the workplace, remains a source of bias due to evolving employment statuses.^3^^,^^4^ Conventional analysis bias when prior exposure affects time-varying confounders leads to the development of processes such as g-methods to counter this.^3^^,^^4^ By primarily focusing on male mortality, these methods, such as g-estimation, have been applied to a few groups of workers.^5–12^ Despite the potential difference in the healthy worker effect by factors such as sex, occupational class and disease causes,^1^^,^^2^ however, scant knowledge exists regarding its modification in female workers. Moreover, few studies have explored the healthy worker effect by using both incidence and mortality data.

Medical radiation workers, constituting over half of all radiation-exposed workers from manmade sources,^13^ have presented direct observations of health effects linked to protracted low-dose radiation exposure.^14^ However, studies on low-dose occupational radiation may face bias due to the low effect estimate.^15^ Moreover, the healthy worker effect could be more pronounced in this group, given their better access to medical care and healthier lifestyles. Medical radiation workers demonstrate lower mortality rates than the general population in various countries.^16–19^ However, little is known about the differences in the healthy worker survivor effect, particularly examining its variation by sex in medical radiation workers, who encompass a larger proportion of females than in other industries.

Therefore, this study was aimed to assess the healthy worker effect on mortality and cancer incidence in a cohort of medical radiation workers stratified by sex. In South Korea, we established a registry-based cohort by merging data from the National Dose Registry (NDR) with national mortality and cancer incidence records. Earlier investigations explored the influence of occupational radiation exposure on overall mortality^19^ and all-cancer incidence in this cohort.^20^ Examination of the healthy worker hire and survivor effects would provide deeper insights into the exposure–response relationship in epidemiological research.

## Methods

### Study population

The study population and methods have been previously detailed.^20^ Briefly, the population encompassed all diagnostic medical radiation workers who were registered in the NDR from 1 January 1996 to 31 December 2011 (*n* = 94 379). This cohort included radiology technologists, radiologists, non-radiologist physicians, dentists, dental hygienists, nurses and medical assistants. After the exclusion of workers with invalid data (*n* = 91) or with a history of cancer before enrolment (*n* = 370), 93 918 workers were included in the analysis. This registry-based linkage cohort study did not involve direct participant contact and received approval from the Institutional Review Board of Korea University.

### Ascertainment of cause of death and cancer incidence

To determine the cause of death among participants, personal identification numbers were sent to Statistics Korea (http://kostat.go.kr/), which linked them to mortality data. Mortality data were categorized based on the underlying cause of death using the International Classification of Diseases and Related Health Problems, 10th Revision (ICD-10). The analysis encompassed all causes of death (A00–Y89), deaths from all malignant neoplasms (C00–C97), solid cancers (C00–C80) and haematopoietic cancers (C81–C96) as defined by using ICD-10 codes.

Cancer incidence was determined by linkage to the Korean Central Cancer Registry (KCCR), a comprehensive national repository that is maintained by the Korean National Cancer Center. The KCCR offers detailed data on cancer codes, sites, histological types, stages and diagnosis dates for cancers that were diagnosed in the cohort members (http://www.ncc.re.kr/). Cancer cases were defined as the first primary malignant tumours, delineated by ICD-10 codes (C00–C97). The same classification of all malignant neoplasms, solid cancers and haematopoietic cancers corresponding to the cause of death was employed by using ICD-10 codes. Additionally, all solid cancers except thyroid cancer were included, given their substantial representation in the total cancers and potential overdiagnosis in South Korea.^21^ Follow-up on diagnostic medical radiation workers for mortality linkage with national vital statistics and cancer incidence continued until 2019 and 2018, respectively.

### Definition of exposure and employment

Occupational exposure data were sourced from the NDR database. Established by the Korean Disease Control and Prevention Agency, the NDR system encompasses all diagnostic medical radiation workers (https://www.kdca.go.kr/). Personal thermoluminescent dosimeters have been utilized for quarterly badge-dose measurements since 1996. The NDR database includes worker details such as name, sex, date of birth, personal identification number, occupation, quarterly recorded dose data, and the start date and end date of the measurement period. Annual and cumulative individual radiation badge doses (measured in Sieverts) based on Hp (10) were computed by consolidating the quarterly badge readings for NDR-enrolled workers.^22^ Occupational radiation exposure was categorized into no exposure (≤1 mSv) and exposure (>1 mSv) groups based on the time-dependent cumulative badge dose of up to each year. A 10-year lag dose was applied, considering the period between radiation exposure and chronic health outcomes. Using NDR data, continuous active employment status was determined between the first and the final badge measurement record dates. Employment status post-2011 was presumed to mirror the 2011 status, the latest linked NDR year.

### Standardized mortality and incidence ratios

Each participant contributed person-years at risk from 1996 or the year of the start of work based on the NDR, whichever occurred later. The end of the follow-up period was defined as the earliest date of death, any diagnosis of cancer, 31 December 2018 (for cancer incidence) or 31 December 2019 (for mortality). By using the DATAB module in Epicure software, a person-year table was generated, stratified by various factors including sex, attained age (<25, 5-year intervals from 25 to 84, ≥85 years), calendar year (1996–2000, 2001–2005, 2006–2010, 2011–2019), birth year (<1960, 5-year intervals from 1960 to 1979, ≥1980), year of job entry (<2000, 2000–2004, ≥2005), job title (physician, non-physician), year first worked (<1996, 1996–2004, ≥2005), age at the first job (<25, 25–29, 30–34, 35–39, ≥40 years), employment duration in years (<1, 1–4, 5–9, ≥10), type of medical facility (general hospital, hospital and clinic, dental hospital and clinic, others) and the location of the medical facility (metropolitan, city, rural). Attained age and calendar year were used as the timescale.

Standardized mortality ratios (SMRs) and standardized incidence ratios (SIRs) were computed by comparing the observed deaths and incident cancers to the expected numbers within specific demographic categories. Expected counts were derived by multiplying the person-years at risk by sex-, age-, and calendar-year-specific mortality rates sourced from South Korean national statistics and cancer incidence rates from the Korean National Cancer Center, employing Poisson regression methods. The analyses were stratified by sex, job title and follow-up period. Sensitivity analyses were performed on workers hired in or after 1996 (*n* = 80 776), when the monitoring programme was started. The analyses were performed using the AMFIT module in Epicure software (version 2.0).

### G-estimation and Weibull regression analysis

**Figure 1.**
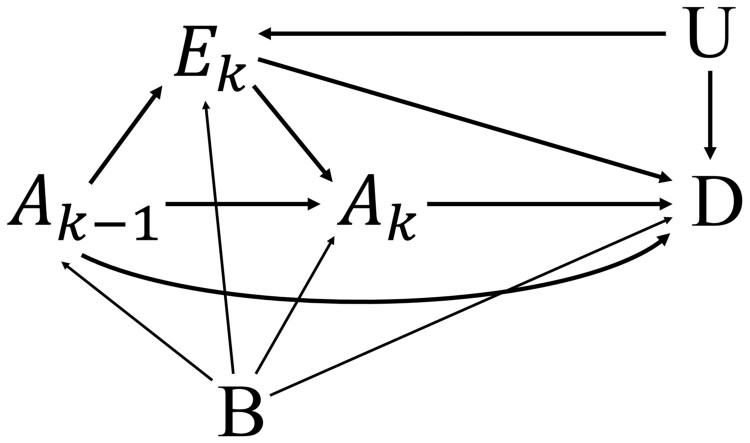
Direct acyclic graph representing healthy worker survivor effect in estimates of the association between occupational radiation exposure and health outcome. $A_{k}$ represents the radiation exposure at time *k* and $A_{k-1}$ represents the prior radiation exposure. $E_{k}$ is a time-varying confounder, employment status (actively employed at time *k*; yes or no). D represents health status (death or cancer incidence). B represents measured baseline variables (e.g. gender, occupation). U represents unmeasured variables (e.g. health status)

A directed acyclic graph was constructed to outline the healthy worker survivor effect (Figure 1). We employed g-estimation to assess the unbiased effects while considering the time-dependent properties of employment status. G-estimation was chosen among g-methods because the inverse probability weighted marginal structural model can introduce bias when exposure does not occur after leaving work and the g-formula is more suitable for estimating intervention effects.^3^^,^^4^ For g-estimation, we applied structural nested accelerated failure time models (SNAFTM) due to their suitability for registry outcomes such as mortality or cancer incidence.^23^

The g-estimation of SNAFTM^24^ is described in detail in the Supplementary material (available as Supplementary data at *IJE* online). To compare the g-estimation results with those generated by using the standard time-varying Weibull regression, we calculated the survival time ratio between always exposed and never exposed through g-estimation. This ratio estimate was then transformed into a hazard ratio (HR) by assuming the Weibull distribution. The Weibull shape parameter from the Weibull regression model, fitted with the same covariates as those in the g-estimation, was utilized to express the causal survival time ratio in proportional hazard parameterization.^25^ For all models, the probability of being uncensored at the end of the follow-up for each individual was estimated via multinomial logistic regression. Inverse probability weights were applied to adjust for competing risk bias.

Sensitivity analyses were conducted on workers who were hired in or after 1996 to mitigate potential left truncation bias. Alternative binary exposure definitions (i.e. 2, 3, 4, 5 mSv) were applied to enhance the robustness of the main findings. Additionally, we performed an analysis that was stratified by sex and job title (physician vs non-physician) by considering the variations in baseline rates and radiation risks. The g-estimation procedures were conducted using the *stgest* and *gesttowb* macros in Stata 17.0 software (StataCorp LLC, College Station, Texas).^26^

## Results

The majority of the diagnostic medical radiation workers in South Korea were born after 1960 and over 70% began working after 1996 (Table 1). The distribution of cumulative badge doses was right-skewed, with 51% of the workers exposed to ≤1 mSv. The mean age at the end of the mortality follow-up period was 47.4 years, with an average follow-up of 15.8 years per worker. Male workers, predominantly physicians, were generally older, started work earlier, had longer tenure and had higher cumulative badge doses than female workers, who were primarily non-physicians, including nurses and assistants.

**Table 1.** Occupational characteristics of diagnostic medical radiation workers in South Korea, stratified by sex

Characteristics	Total		Male		Female	
	Number	%	Number	%	Number	%
Total	93 918	100.0	53 581	57.1	40 337	42.9
Occupation						
Physician	35 680	38.0	29 148	54.4	6532	16.2
Non-physician	58 238	62.0	24 433	45.6	33 805	83.8
Type of facility						
General hospital	21 125	22.5	11 254	21.0	9871	24.5
Hospital and clinic	36 587	39.0	26 390	49.2	10 197	25.2
Dental hospital and clinic	31 769	33.8	12 414	23.2	19 355	48.0
Others	4437	4.7	3523	6.6	914	2.3
Area of facility						
Metropolitan	50 067	53.3	26 534	49.5	23 533	58.3
City	37 984	40.4	22 549	42.1	15 435	38.3
Rural	5867	6.3	4498	8.4	1369	3.4
Calendar year of birth						
<1960	10 488	11.1	9623	18.0	865	2.2
1960–1964	10 489	11.2	8714	16.3	1775	4.4
1965–1969	13 855	14.8	10 054	18.7	3801	9.4
1970–1974	16 132	17.2	9740	18.2	6392	15.8
1975–1979	16 762	17.8	7298	13.6	9464	23.5
≥1980	26 192	27.9	8152	15.2	18 040	44.7
Age at entry (years)						
<25	30 388	32.4	10 904	20.4	19 484	48.3
25–29	25 957	27.6	14 726	27.4	11 231	27.8
30–34	13 922	14.8	9090	17.0	4832	12.0
35–39	11 477	12.2	8678	16.2	2799	7.0
≥40	12 174	13.0	10 183	19.0	1991	4.9
Calendar year in which work began						
<1996	13 146	14.0	9818	18.3	3328	8.3
1996–2004	35 123	37.4	21 718	40.6	13 405	33.2
≥2005	45 649	48.6	22 045	41.1	23 604	58.5
Attained age (mean ± 2 SD)						
Mortality	47.4 ± 20.8		51.2 ± 21.2		42.3 ± 15.2	
Cancer incidence	46.2 ± 20.6		50.0 ± 21.0		41.1 ± 15.1	
Follow-up period (year, mean ± 2SD)						
Mortality	15.8 ± 10.2		16.6 ± 10.5		14.7 ± 9.5	
Cancer incidence	14.6 ± 10.4		15.4 ± 10.6		13.5 ± 9.6	
Duration of employment (years)						
<1	18 132	19.3	7425	13.9	10 707	26.5
1–4	33 025	35.2	15 692	29.3	17 333	43.0
5–9	22 103	23.5	14 012	26.2	8091	20.1
≥10	20 658	22.0	16 452	30.6	4206	10.4
Cumulative badge dose (mSv)						
≤ 1	48 091	51.2	22 039	41.1	26 052	64.5
1–5	21 230	22.6	12 369	23.1	8861	22.0
5–20	14 963	15.9	10 501	19.6	4462	11.1
≥20	9634	10.3	8672	16.2	962	2.4

In total, 1831 deaths and 3759 first primary cancer cases were reported in the cohort during the study period (Table 2). SMR analysis of all-cause mortality revealed a substantial deficit for male (SMR = 0.44; 95% CI: 0.42, 0.46) and female workers (SMR = 0.53; 95% CI: 0.46, 0.60) compared with the national rates. Whereas male workers showed a lower risk of solid cancer incidence than the general South Korean population (SIR = 0.88; 95% CI: 0.84, 0.92), female workers showed an elevated risk (SIR = 1.11; 95% CI: 1.05, 1.17). The exclusion of thyroid cancers decreased the SIRs in both sexes. Stratified analyses by follow-up year showed the highest SMRs and SIRs in workers with less than a 10-year follow-up, which levelled off in subsequent periods, except for haematopoietic cancers (Supplementary Table S1, available as Supplementary data at *IJE* online). The findings were similar between physicians and non-physicians after stratification by sex (Table 3). Similar findings were observed when analysing workers who started their jobs in or after 1996 (Supplementary Table S2, available as Supplementary data at *IJE* online).

**Table 2.** Standardized mortality ratio of causes of death and standardized incidence ratio of cancers among South Korean medical diagnostic radiation workers, stratified by sex

Cause of death (ICD-10 codes)	Total		Male		Female	
	Observed cases	SMR (95% CI)	Observed cases	SMR (95% CI)	Observed cases	SMR (95% CI)
All causes of death (A00–Y89)	1831	0.45 (0.43, 0.47)	1604	0.44 (0.42, 0.46)	227	0.53 (0.46, 0.60)
All malignant neoplasms (C00–C97)	717	0.60 (0.55, 0.64)	615	0.58 (0.53, 0.63)	102	0.71 (0.57, 0.85)
Solid cancers (C00–C80)	660	0.58 (0.54, 0.63)	562	0.56 (0.52, 0.61)	98	0.74 (0.60, 0.89)
Haematopoietic cancers (C81–C96)	55	0.77 (0.59, 1.01)	51	0.86 (0.62, 1.09)	4	0.34 (0.11, 0.79)
**Cancer incidence (ICD-10 codes)**	**Observed cases**	**SIR (95% CI)**	**Observed cases**	**SIR (95% CI)**	**Observed cases**	**SIR (95% CI)**
All malignant neoplasms (C00–C97)	3759	0.97 (0.94, 1.01)	2313	0.90 (0.87, 0.94)	1446	1.11 (1.05, 1.16)
Solid cancers (C00–C80)	3548	0.96 (0.93, 0.99)	2137	0.88 (0.84, 0.92)	1411	1.11 (1.05, 1.17)
Solid cancers other than thyroid	2485	0.80 (0.77, 0.83)	1760	0.75 (0.72, 0.79)	725	0.94 (0.87, 1.01)
Haematopoietic cancers (C81–C96)	185	1.15 (1.00, 1.33)	156	1.26 (1.07, 1.46)	29	0.79 (0.50, 1.07)

SMR, standardized mortality ratio; SIR, standardized incidence ratio; ICD-10, International Classification of Diseases and Related Health Problems, 10th Revision.

**Table 3:** Standardized mortality ratio of causes of death and standardized incidence ratio of cancers among South Korean medical diagnostic radiation workers, classified by job titles and sex

Cause of death (ICD-10 codes)	Male				Female			
	Physicians		Non-physicians		Physicians		Non-physicians	
	Observed cases	SMR (95% CI)	Observed cases	SMR (95% CI)	Observed cases	SMR (95% CI)	Observed cases	SMR (95% CI)
All causes of death (A00–Y89)	915	0.40 (0.37, 0.42)	689	0.52 (0.48, 0.56)	58	0.50 (0.37, 0.63)	169	0.54 (0.46, 0.62)
All malignant neoplasms (C00–C97)	381	0.54 (0.49, 0.60)	234	0.66 (0.57, 0.74)	27	0.61 (0.38, 0.84)	75	0.75 (0.58, 0.92)
Solid cancers (C00–C80)	347	0.52 (0.47, 0.58)	215	0.64 (0.56, 0.73)	26	0.63 (0.39, 0.88)	72	0.79 (0.61, 0.98)
Haematopoietic cancers (C81–C96)	33	0.88 (0.58, 1.18)	18	0.82 (0.44, 1.19)	1	0.33 (null, 1.46)	3	0.34 (0.09, 0.89)
**Cancer incidence (ICD-10 codes)**	**Observed cases**	**SIR (95% CI)**	**Observed cases**	**SIR (95% CI)**	**Observed cases**	**SIR (95% CI)**	**Observed cases**	**SIR (95% CI)**
All malignant neoplasms (C00–C97)	1504	0.90 (0.86, 0.95)	809	0.91 (0.85, 0.97)	367	1.15 (1.03, 1.27)	1079	1.09 (1.03, 1.16)
Solid cancers (C00–C80)	1399	0.88 (0.83, 0.92)	738	0.88 (0.82, 0.94)	354	1.14 (1.02, 1.26)	1057	1.10 (1.04, 1.17)
Solid cancers other than thyroid	579	0.76 (0.72, 0.81)	1181	0.73 (0.67, 0.79)	219	1.06 (0.93, 1.21)	506	0.90 (0.82, 0.98)
Haematopoietic cancers (C81–C96)	91	1.22 (0.97, 1.47)	65	1.33 (1.01, 1.65)	9	1.13 (0.54, 2.04)	20	0.69 (0.39, 0.99)

SMR, standardized mortality ratio; SIR, standardized incidence ratio; ICD-10, International Classification of Diseases and Related Health Problems, 10th Revision.

Table 4 displays estimates for the causes of death and cancer incidence obtained by using g-estimation and Weibull regression categorized by sex. Among male workers, g-estimated HRs were higher for all causes of death (HR = 2.55; 95% CI: 1.97, 3.23) and cancer incidence (HR = 1.96; 95% CI: 1.52, 2.55) compared with Weibull estimates (HR = 1.26; 95% CI: 0.86, 1.86; HR = 1.34; 95% CI: 0.99, 1.83, respectively). However, the changes for female workers were somewhat opposite to those of male workers. The disparity between Weibull regression and g-estimation was more pronounced for cancer deaths than for cancer incidences in both male and female workers. Similar patterns were observed between physicians and non-physicians after stratification by sex (Table 5). Furthermore, the findings were similar when alternative exposure definitions were applied (Supplementary Table S3, available as Supplementary data at *IJE* online) and when analysing workers who started their jobs in or after 1996 (Supplementary Table S4, available as Supplementary data at *IJE* online).

**Table 4.** Occupational radiation exposure and the risks of mortality and cancer incidence using Weibull regression and G-estimation among South Korean diagnostic medical radiation workers, stratified by sex

	Total		Male		Female	
	Weibull regression	G-estimation	Weibull regression	G-estimation	Weibull regression	G-estimation
	HR^a^ (95% CI)	HR^a^ (95% CI)	HR^a^ (95% CI)	HR^a^ (95% CI)	HR^a^ (95% CI)	HR^a^ (95% CI)
**Cause of death (ICD-10 codes)**						
All causes of death (A00–Y89)	1.38 (0.98, 1.95)	2.34 (1.70, 2.91)	1.26 (0.86, 1.86)	2.55 (1.97, 3.23)	2.12 (0.97, 4.62)	0.72 (0.40, 3.69)
All malignant neoplasms (C00–C97)	0.97 (0.54, 1.76)	1.97 (0.82, 2.76)	0.95 (0.48, 1.88)	2.33 (1.59, 3.29)	1.18 (0.35, 4.02)	0.28 (0.12, 0.73)
Solid cancers (C00–C80)	1.05 (0.58, 1.91)	2.12 (0.81, 3.24)	1.06 (0.53, 2.11)	2.54 (1.79, 3.45)	1.17 (0.34, 4.02)	0.27 (0.11, 0.73)
Haematopoietic cancers (C81–C96)	1.73 (0.64, 4.62)	1.75 (1.00, 2.44)	1.73 (0.64, 4.62)	1.82 (1.00, 2.44)	NC	NC
**Cancer incidence (ICD-10 codes)**						
All malignant neoplasms (C00–C97)	1.30 (1.03, 1.64)	1.62 (1.34, 2.04)	1.34 (0.99, 1.83)	1.96 (1.52, 2.55)	1.27 (0.88, 1.82)	0.83 (0.59, 1.65)
Solid cancers (C00–C80)	1.30 (1.03, 1.66)	1.63 (1.37, 2.08)	1.37 (1.00, 1.88)	2.08 (1.52, 2.86)	1.24 (0.85, 1.80)	0.83 (0.59, 1.65)
Solid cancers other than thyroid	1.11 (0.82, 1.50)	1.51 (1.28, 2.02)	1.27 (0.89, 1.81)	2.01 (1.39, 2.79)	0.84 (0.46, 1.54)	0.71 (0.44, 1.48)
Haematopoietic cancers (C81–C96)	0.95 (0.28, 3.27)	1.55 (0.39, 2.54)	0.44 (0.06, 3.44)	1.66 (0.34, 2.94)	0.63 (0.15, 2.66)	0.65 (0.43, 3.79)

HR, hazard ratio; ICD-10, International Classification of Diseases and Related Health Problems, 10th Revision; NC, non-convergence.

a Adjusted for employment status (binary), attained age (continuous), sex, birth year (<1960, 5-year intervals from 1960 to 1979, ≥1980) and years of employment duration (<1, 1–4, 5–9, ≥10).

**Table 5.** Occupational radiation exposure and the risks of mortality and cancer incidence using Weibull regression and G-estimation among South Korean diagnostic medical radiation workers, classified by job titles and sex

	Male				Female			
	Physicians		Non-physicians		Physicians		Non-physicians	
	Weibull regression	G-estimation	Weibull regression	G-estimation	Weibull regression	G-estimation	Weibull regression	G-estimation
	HR^a^ (95% CI)	HR^a^ (95% CI)	HR^a^ (95% CI)	HR^a^ (95% CI)	HR^a^ (95% CI)	HR^a^ (95% CI)	HR^a^ (95% CI)	HR^a^ (95% CI)
**Cause of death (ICD-10 codes)**								
All causes of death (A00–Y89)	0.92 (0.54, 1.55)	2.15 (1.31, 2.92)	1.95 (1.01, 3.66)	2.50 (1.49, 5.10)	0.66 (0.08, 5.29)	0.46 (0.25, 4.95)	3.14 (1.32, 7.48)	0.76 (0.30, 10.62)
All malignant neoplasms (C00–C97)	0.78 (0.34, 1.81)	1.98 (0.87, 3.02)	1.37 (0.39, 4.85)	2.26 (0.95, 3.31)	0.33 (0.09, 1.18)	0.32 (0.16, 2.91)	0.48 (0.20, 1.15)	0.48 (0.12, 0.72)
Solid cancers (C00–C80)	0.87 (0.37, 2.01)	2.30 (0.79, 3.87)	1.42 (0.39, 5.10)	2.48 (1.25, 3.30)	0.32 (0.09, 1.18)	0.32 (0.16, 2.91)	0.48 (0.20, 1.15)	0.48 (0.12, 0.72)
Haematopoietic cancers (C81–C96)	1.44 (0.58, 3.56)	1.85 (0.63, 2.13)	NC	NC	NC	NC	NC	NC
**Cancer incidence (ICD-10 codes)**								
All malignant neoplasms (C00–C97)	1.29 (0.90, 1.84)	1.66 (1.27, 2.22)	1.53 (0.80, 2.94)	1.92 (0.85, 8.13)	0.70 (0.28, 1.73)	1.44 (0.50, 2.40)	1.50 (1.00, 2.24)	0.84 (0.59, 1.86)
Solid cancers (C00–C80)	1.28 (0.89, 1.83)	1.62 (1.23, 2.23)	1.76 (0.91, 3.40)	3.30 (1.37, 9.23)	0.59 (0.21, 1.62)	1.47 (0.48, 2.48)	1.49 (0.99, 2.23)	0.83 (0.58, 1.86)
Solid cancers other than thyroid	1.21 (0.81, 1.80)	1.55 (1.18, 2.24)	1.61 (0.73, 3.57)	3.24 (1.14, 10.68)	0.57 (0.34, 0.95)	1.42 (0.37, 2.28)	1.37 (0.73, 2.57)	0.70 (0.41, 1.53)
Haematopoietic cancers (C81–C96)	0.78 (0.10, 6.34)	1.80 (1.00, 3.76)	0.61 (0.21, 1.82)	0.78 (0.10, 1.58)	NC	NC	0.46 (0.05, 3.97)	0.65 (0.38, 1.79)

HR, hazard ratio; ICD-10, International Classification of Diseases and Related Health Problems, 10th Revision; NC, non-convergence.

a Adjusted for employment status (binary), attained age (continuous), sex, birth year (<1960, 5-year intervals from 1960 to 1979, ≥1980) and years of employment duration (<1, 1–4, 5–9, ≥10).

## Discussion

Our findings report both healthy worker hire and survivor effects within a cohort of diagnostic medical radiation workers. These effects displayed a greater impact on mortality data than incidence data and were different in male and female workers. The findings were consistent across sensitivity analyses. Our results indicate a potential underestimation of the effect of occupational radiation exposure in mortality studies among male workers, highlighting the need to consider the healthy worker survivor effect. The different patterns of the healthy worker effects in female workers emphasize the importance of considering this effect according to sex in epidemiological studies.

The lower SMRs in this study compared with that of the general population reflect the healthy worker hire effect and are consistent with previous studies on medical radiation workers in the USA,^16^ Canada^17^ and France.^18^ Our SIR findings are also largely in line with studies on US radiologic technologists^27^ and male Canadian medical radiation workers.^17^ The decrease in SIRs when thyroid cancer was excluded in both male and female workers supports the easier cancer-screening access in the employed population.^28^

The HRs from g-estimation revealed a higher risk, by 51% for all deaths and 32% for all-cancer incidences in male workers, compared with the standard model. Similar differences were observed in previous studies, such as a 35% stronger effect estimate of lung cancer mortality among textile factory workers exposed to asbestos^11^ and a 39% increase in the radon-lung cancer mortality association in Colorado Plateau uranium miners^8^ when accounting for time-varying confounders affected by prior exposure. The different results from g-estimation and Weibull regression likely stem from the healthy worker survivor effect in this population. This effect operates through a continuous selection process, favouring the retention of healthier individuals in employment compared with those who exit, often leading to an attenuation of exposure–response curves in occupational cohort studies.^29^ Poor health increased the risk of exit from paid employment due to disability pension, unemployment and early retirement in a systematic review.^30^ Our findings, showing the lowest SMRs and SIRs with longer follow-up periods, support this trend. Despite the various occupational hazards that are faced by medical radiation workers in hospitals,^31^^,^^32^ factors linked to employment, such as better access to healthcare and improved living conditions, may have beneficial effects. However, the differentiated roles of health in the transition from work to retirement^33^ warrant further investigation.

Methodological disparities between the Weibull regression and g-estimation, such as parametric vs semi-parametric models and the approach to control confounding, may also contribute to the difference. G-estimation provides marginal estimates, comparing health outcomes that are experienced by the entire population under specific exposure scenarios, whereas the Weibull models offer conditional associations, comparing health outcomes between subgroups with different exposures.^5^ Therefore, a direct comparison of the estimates between the Weibull regression and g-estimation should be cautiously interpreted.

The different healthy worker survivor bias patterns in male and female workers can be attributed to their occupational and social circumstances. Notably, some healthy women often leave the workforce for reasons that are unrelated to health, such as childcare responsibilities^34^, a trend that is less common among men. The healthy worker survivor effect was weak among US women,^6^ whereas the European low-skilled production female workers showed a stronger effect than male workers.^35^ Women who work outside the home were more likely than women working in the home to have poor health and less healthy lifestyles in Japan.^36^ In our cohort, most female workers were young professionals, potentially contributing to an opposite healthy worker survival effect to that of male workers. With the rising proportion of female workers, the understanding of gender-specific disparities in the healthy worker effect necessitates further investigation.

Our findings highlight a greater impact of healthy worker hire and survivor effects on mortality than on incidence. The disparity observed in the SMRs for deaths compared with SIRs for cancer incidence compared with the general population was similar to that observed in other occupational cohort studies,^37^^,^^38^ indicating a strong healthy hire effect on mortality. The differences in risk estimates between g-estimation and Weibull regression were also more pronounced for cancer mortality than for cancer incidence in both male and female workers. This could be because more factors that are associated with mortality impact the employment selection process and continued work within the industry. For instance, medical workers might benefit from early diagnosis and extended survival due to their occupation, influencing mortality rates differently than disease incidence.

The consistency in these findings when analysing workers hired in or after 1996 suggests a lack of the noticeable left truncation bias that is often observed in occupational cohort studies; workers less susceptible to exposure tend to remain exposed for longer durations.^39^ This could be influenced by the relatively low proportion of workers who started their jobs before the inception of the monitoring programme in 1996, accounting for only 13% of the total participants. Our study found no distinctive disparity in the healthy worker effect between the physician and non-physician groups, although non-physicians tended to have higher SMRs, potentially attributed to the relatively homogeneous characteristics within our cohort of hospital workers or the effect from job difference has not yet contributed sufficiently to the follow-up period. The observation of a less pronounced healthy worker effect for cancer compared with non-cancer diseases also aligns with typical trends in occupational epidemiology.^2^

The strength of this study lies in its comprehensive assessment of both healthy worker hire and survivor effects by using mortality and incidence data that are categorized by sex. To the best of our knowledge, this is the first application of g-estimation to mitigate the healthy survivor effect among medical radiation workers. Moreover, the study benefits from linkages to comprehensive national mortality and cancer incidence registries that encompass all monitored diagnostic medical radiation workers in South Korea. The utilization of quarterly dosimetry data as a time-varying surrogate for employment status in the g-estimation is another advantage.

However, this study has some limitations. First, the cumulative binary exposure definition that was employed may have led to potential exposure misclassification and residual healthy worker survivor bias, warranting consideration of more relevant exposure metrics in future studies. Second, the lack of complete employment status data after 2011 among cohort members necessitates further investigations with updated cohort information. Third, the results are only valid under the assumption of consistency, no interference, correct model specification and conditional exchangeability.^23^

In conclusion, our findings highlight the importance of addressing the healthy worker effect in occupational cohort studies. Considering the relatively low risk from low-dose radiation exposure, understanding of its impact on the healthy worker effect becomes essential when assessing health risks among radiation workers. Our investigation also shows notable distinctions of healthy worker effect by sex, shedding light on the need for sex-specific considerations in future research. Moreover, the use of incidence data is advantageous in mitigating the influence of the healthy worker effect. Recognition of the dynamic nature of the healthy worker effect, influenced by multiple factors over time, is important for comprehension of the exposure–response relationships in epidemiological studies.

## Ethics approval

This study was approved by the Institutional Review Board of Korea University (KUIRB-2019–0092-08).

## Supplementary Material

dyae130_Supplementary_Data

## Data Availability

No data are available.
