# Supplementary material for: Healthy worker hire and survivor effects in a cohort of medical radiation workers
Source: Int J Epidemiol. 2024 Oct 4;53(5):dyae130. doi: 10.1093/ije/dyae130 (PMC11452196; doi:10.1093/ije/dyae130)
Supplement: dyae130_Supplementary_Data [file dyae130_supplementary_data.docx]

**Supplementary material**. Technical details for the G-estimation of structural nested accelerated failure-time models

$T\left( \psi\right)$ denotes the function of the potential survival time for individuals who were never exposed, with $\psi$ representing the unknown parameter to be estimated. It was based on the structural accelerated failure time model, which establishes a relationship between the observed survival time of each individual and the counterfactual survival time:

$T\left( \psi\right)=\int_{0}^{T_{\bar{A}}} e^{\psi A_{k}} dk$

where $A_{k}$ denotes the dichotomization of observed radiation exposure at time *k,* and a candidate value of $\psi$ is assigned. $\bar{A}=[A_{1}, A_{2}, A_{3},\ldots]$represents a time-varying treatment regime for each subject and $T_{\bar{A}}$ represents the survival time under the treatment regime $\bar{A}$. G-estimation involves a two-step iterative process to estimate the true parameter $\psi$ (denoted as $\psi^{*}$) and compute $T\left( \psi^{*} \right)$, which signifies the counterfactual survival time that would have been observed if individuals had never been exposed.

In the first step, a candidate value of $\psi$ was selected and $T\left( \psi\right)$ was calculated for each subject. When the true value of the parameter is used, the function represents survival times that would have been observed had individuals never been exposed (denoted as $T_{\bar{0}}$):

$$T_{\bar{0}}= T\left( \psi^{*} \right)=\int_{0}^{T_{\bar{A}}} e^{\psi^{*}A_{k}} dk$$

In the second step, the pooled logistic model equation was employed to analyze the association between $T\left( \psi\right)$ and the observed exposure $A_{k}$ at each time point $k$ to identify the true parameter value for the candidate $\psi$:

$logit Pr\left[ A_{k}=1 | T\left( \psi\right), \bar{A}_{k-1},\bar{E}_{k}, X \right]=\beta_{0}+\beta_{1}T\left( \psi\right)+\bar{A}_{k-1}\beta_{2}+\beta_{3}E_{k}+\bar{E}_{k-1}\beta_{4}+X\beta_{5}$

where $\bar{A}_{k-1}$ denotes the exposure history, including the set of exposure at the previous period and baseline effect, $\bar{E}_{k-1}$ denotes the employment status history that represents the set of employment status at the previous period and baseline effect of employment status, $X$ is a vector of confounding factors like attained age, sex, year of birth, and years of employment duration. This step aims to identify the parameter value (i.e. $\psi^{*}$) that ensures $T\left( \psi\right)$ remains independent of $A_{k}$. If $\psi$ is the value for which the Wald statistic for $\beta_{1}$ equals zero in the pooled logistic regression model, the estimate of the true parameter $\psi^{*}$ was employed. The upper and lower bounds of the 95% CIs for $\psi^{*}$ were determined by the Wald statistic of $\beta_{1}$.

The two steps were iteratively executed using an interval bisection search algorithm around the $\psi$ candidate until the value of $\psi$ that makes $T\left( \psi\right)$ independent of $A_{k}$ was identified.^26^ The value $e^{{-\psi}^{*}}$ is the expansion factor in survival time when comparing always exposed versus never exposed, i.e. $e^{{-\psi}^{*}}=T_{\bar{A}}/T_{\bar{0}}$ for $\bar{A}=[1, 1, 1,\ldots]$, called as causal survival time ratio. Under the assumption of no unmeasured confounders and correct model specification, $e^{{-\psi}^{*}}=1 (\psi^{*}=0$) represents the null hypothesis, $e^{{-\psi}^{*}}>1 (\psi^{*}<0$) indicates that exposure increase time to the outcome event, and $e^{{-\psi}^{*}}<1 (\psi^{*}>0$) indicates that exposure decrease the time to survive.

To compare the g-estimation results with those generated by the standard time-varying Weibull regression, we calculated the causal survival time ratio between always exposed and never exposed as ${\exp(-\psi}^{*})$ under the parameter $\psi^{*}$ obtained through g-estimation. This study transformed this ratio estimate into hazard ratios to express the framework of proportional hazards parameterization, given that the prevailing parameterization for survival analysis in epidemiology is proportional hazards. One way to transform is by utilizing the Weibull distribution that can be expressed in either parameterization.^26^ The regression model based on the Weibull distribution offers the advantage of interpretations from two approaches, as it can be constructed from both accelerated failure time and proportional hazards parameterizations. Weibull accelerated failure time model yields a survival time ratio based on the vector of coefficients for each covariate (defined as $\alpha$) and the shape parameter (defined as $\sigma$). The Weibull shape parameter can be utilized to express results from the accelerated failure time parameterization as proportional hazards: $\beta=-\alpha/\sigma$, where $\beta$ has a hazard ratio interpretation. If the underlying survival times are assumed to follow a Weibull distribution, the Weibull shape parameter can, therefore, be used to express the G-estimated survival ratio as a hazard ratio for the exposure.^25,26^

**Table S1.** Standardized mortality ratio of causes of death and standardized incidence ratio of cancers among South Korean diagnostic medical radiation workers during the follow-up period

|  | |  | SMR (95% CI) by follow-up period (year) | | | | |
| --- | --- | --- | --- | --- | --- | --- | --- |
| Cause of death (ICD-10 codes) | SMR (95% CI) |  | | $\leq$ 10  (N=14 638) | 10 – 15  (N=31 871) | 15 – 20  (N=24 772) | > 20  (N=22 637) |
| All causes of death (A00-Y89) | 0.45 (0.43, 0.47) |  | | 0.80 (0.65, 0.99) | 0.45 (0.41, 0.50) | 0.50 (0.46, 0.54) | 0.40 (0.38, 0.43) |
| Cases | 1831 |  | | 91 | 394 | 549 | 797 |
| All malignant neoplasms (C00-C97) | 0.60 (0.55, 0.64) |  | | 0.95 (0.66, 1.37) | 0.59 (0.50, 0.69) | 0.67 (0.58, 0.76) | 0.54 (0.48, 0.60) |
| Cases | 717 |  | | 29 | 148 | 216 | 324 |
| Solid cancers (C00-C80) | 0.58 (0.54, 0.63) |  | | 0.96 (0.66, 1.40) | 0.57 (0.48, 0.67) | 0.65 (0.57, 0.75) | 0.54 (0.48, 0.60) |
| Cases | 660 |  | | 27 | 133 | 198 | 302 |
| Hematopoietic cancers (C81-C96) | 0.77 (0.59, 1.01) |  | | 0.92 (0.15, 2.84) | 0.91 (0.55, 1.52) | 0.93 (0.59, 1.48) | 0.60 (0.39, 0.93) |
| Cases | 55 |  | | 2 | 15 | 18 | 20 |
|  |  |  | | SIR (95% CI) by follow-up period (year) | | | |
| Cancer incidence (ICD-10 codes) | SIR (95% CI) |  | | $\leq$ 10  (N=21 775) | 10 – 15  (N=31 470) | 15 – 20  (N=21 727) | > 20  (N=18 946) |
| All malignant neoplasms (C00-C97) | 0.97 (0.94, 1.01) |  | | 1.28 (0.16, 1.42) | 1.01 (0.95, 1.08) | 0.96 (0.90, 1.01) | 0.89 (0.84, 0.94) |
| Cases | 3759 |  | | 389 | 985 | 1202 | 1183 |
| Solid cancers (C00-C80) | 0.96 (0.93, 0.99) |  | | 1.29 (1.16, 1.42) | 0.99 (0.93, 1.05) | 0.94 (0.89, 1.00) | 0.88 (0.83, 0.93) |
| Cases | 3548 |  | | 373 | 922 | 1,134 | 1119 |
| Solid cancers other than thyroid | 0.80 (0.77, 0.83) |  | | 1.08 (0.95, 1.23) | 0.82 (0.76, 0.89) | 0.79 (0.74, 0.85) | 0.75 (0.70, 0.80) |
| Cases | 2485 |  | | 225 | 598 | 790 | 872 |
| Hematopoietic cancers (C81-C96) | 1.15 (1.00, 1.33) |  | | 1.19 (0.71, 1.97) | 1.37 (1.05, 1.79) | 1.16 (0.90, 1.50) | 0.98 (0.76, 1.28) |
| Cases | 185 |  | | 15 | 55 | 60 | 55 |

SMR, standardized mortality ratio; SIR, standardized incidence ratio; N, total number of cases in each period; CI, confidence interval; ICD-10, International Classification of Diseases and Related Health Problems, 10th Revision.

**Table S2.** Standardized mortality ratio of causes of death and standardized incidence ratio of cancers among South Korean medical diagnostic radiation workers who started job ≥1996, stratified by sex

|  | Total | |  | Male | | Female | |
| --- | --- | --- | --- | --- | --- | --- | --- |
| Cause of death (ICD-10 codes) | Observed cases | SMR (95% CI) |  | Observed cases | SMR (95% CI) | Observed cases | SMR (95% CI) |
| All causes of death (A00-Y89) | 1139 | 0.46 (0.43, 0.49) |  | 956 | 0.45 (0.42, 0.47) | 183 | 0.53 (0.46, 0.61) |
| All malignant neoplasms (C00-C97) | 422 | 0.59 (0.54, 0.65) |  | 343 | 0.57 (0.51, 0.63) | 79 | 0.72 (0.56, 0.87) |
| Solid cancers (C00-C80) | 384 | 0.57 (0.52, 0.63) |  | 309 | 0.54 (0.48, 0.60) | 75 | 0.74 (0.58, 0.91) |
| Hematopoietic cancers (C81-C96) | 38 | 0.81 (0.59, 1.12) |  | 34 | 0.91 (0.60, 1.22) | 4 | 0.43 (0.13, 1.00) |
| Cancer incidence (ICD-10 codes) | Observed cases | SIR (95% CI) |  | Observed cases | SIR (95% CI) | Observed cases | SIR (95% CI) |
|  |  |  |  |  |  |  |  |
| All malignant neoplasms (C00-C97) | 2671 | 0.99 (0.96, 1.03) |  | 1505 | 0.94 (0.89, 0.99) | 1166 | 1.07 (1.01, 1.14) |
| Solid cancers (C00-C80) | 2519 | 0.98 (0.94, 1.02) |  | 1380 | 0.91 (0.86, 0.96) | 1139 | 1.08 (1.02, 1.14) |
| Solid cancers other than thyroid | 1644 | 0.80 (0.76, 0.84) |  | 1088 | 0.76 (0.72, 0.81) | 556 | 0.90 (0.82, 0.97) |
| Hematopoietic cancers (C81-C96) | 135 | 1.19 (1.00, 1.40) |  | 112 | 1.35 (1.10, 1.60) | 23 | 0.74 (0.44, 1.05) |

SMR, standardized mortality ratio; SIR, standardized incidence ratio; CI, confidence interval; ICD-10, International Classification of Diseases and Related Health Problems, 10th Revision.

**Table S3.** Occupational radiation exposure and the risks of mortality and cancer incidence using Weibull regression and G-estimation among South Korean diagnostic medical radiation workers applying 3 mSv as alternative exposure definition, stratified by sex

|  | Total | |  | Male | | Female | |
| --- | --- | --- | --- | --- | --- | --- | --- |
|  | Weibull Regression | G-estimation |  | Weibull Regression | G-estimation | Weibull Regression | G-estimation |
| Cause of death (ICD-10 codes) | HR^a^  (95% CI) | HR^a^  (95% CI) |  | HR^a^  (95% CI) | HR^a^  (95% CI) | HR^a^  (95% CI) | HR^a^  (95% CI) |
| All causes of death (A00-Y89) | 1.26  (0.82, 1.94) | 1.48  (1.18, 1.98) |  | 1.32  (0.83, 2.07) | 1.78  (1.31, 2.43) | 0.86  (0.21, 3.52) | 0.85  (0.38, 1.66) |
| All malignant neoplasms (C00-C97) | 1.64  (0.90, 2.97) | 1.72  (0.89, 2.72) |  | 1.84  (0.98, 3.45) | 2.13  (1.31, 5.09) | 0.77  (0.10, 5.87) | 0.69  (0.26, 2.24) |
| Solid cancers (C00-C80) | 1.30  (0.65, 2.57) | 1.56  (0.87, 2.52) |  | 1.44  (0.70, 2.98) | 1.95  (1.10, 4.87) | 0.77  (0.10, 5.87) | 0.69  (0.18, 2.12) |
| Hematopoietic cancers (C81-C96) | 1.96  (0.75, 5.16) | 1.66  (0.87, 3.36) |  | 1.96  (0.74, 5.16) | 1.69  (0.85, 2.14) | NC | NC |
| Cancer incidence (ICD-10 codes) |  |  |  |  |  |  |  |
| All malignant neoplasms (C00-C97) | 1.28  (0.97, 1.69) | 1.24  (0.92, 1.46) |  | 1.14  (0.79, 1.66) | 1.39  (1.12, 1.65) | 1.53  (1.01, 2.30) | 0.84  (0.69, 1.29) |
| Solid cancers (C00-C80) | 1.34  (1.01, 1.77) | 1.21  (0.88, 1.45) |  | 1.24  (0.85, 1.81) | 1.36  (1.06, 1.73) | 1.50  (0.98, 2.28) | 0.84  (0.68, 1.30) |
| Solid cancers other than thyroid | 1.24  (0.87, 1.77) | 1.26  (1.06, 1.55) |  | 1.30  (0.86, 1.98) | 1.42  (1.14, 2.06) | 1.15  (0.60, 2.21) | 0.85  (0.67, 1.39) |
| Hematopoietic cancers (C81-C96) | 0.42  (0.06, 3.03) | 1.43  (0.77, 2.37) |  | 1.28  (0.71, 2.31) | 1.55  (1.13, 2.97) | 0.77  (0.20, 3.00) | 0.64  (0.48, 0.88) |

HR, hazard ratio; CI, confidence interval; ICD-10, International Classification of Diseases and Related Health Problems, 10th Revision; NC, nonconvergence.

^a^Adjusted for employment status (binary), attained age (continuous), sex, birth year (<1960, 5-year intervals from 1960 to 1979, ≥1980), and years of employment duration (<1, 1-4, 5-9, ≥10).

**Table S4.** Occupational radiation exposure and the risks of mortality and cancer incidence using Weibull regression and G-estimation among South Korean diagnostic medical radiation workers who started job ≥1996, stratified by sex

|  | Total | |  | Male | | Female | |
| --- | --- | --- | --- | --- | --- | --- | --- |
|  | Weibull Regression | G-estimation |  | Weibull Regression | G-estimation | Weibull Regression | G-estimation |
| Cause of death (ICD-10 codes) | HR^a^  (95% CI) | HR^a^  (95% CI) |  | HR^a^  (95% CI) | HR^a^  (95% CI) | HR^a^  (95% CI) | HR^a^  (95% CI) |
| All causes of death (A00-Y89) | 1.27  (0.87, 1.85) | 2.52  (1.84, 3.43) |  | 1.11  (0.73, 1.71) | 2.68  (1.98, 3.39) | 2.26  (0.97, 5.25) | 0.80  (0.54, 7.46) |
| All malignant neoplasms (C00-C97) | 1.05  (0.56, 1.99) | 2.09  (0.85, 3.35) |  | 0.98  (0.47, 2.05) | 2.15  (1.38, 3.19) | 1.50  (0.42, 5.38) | 0.46  (0.17, 1.00) |
| Solid cancers (C00-C80) | 1.16  (0.61, 2.21) | 2.33  (0.85, 3.68) |  | 1.10  (0.52, 2.31) | 2.54  (1.49, 3.37) | 1.53  (0.42, 5.51) | 0.46  (0.17, 6.54) |
| Hematopoietic cancers (C81-C96) | 0.80  (0.24, 2.63) | 0.55  (0.20, 0.82) |  | 0.80  (0.24, 2.63) | 0.55  (0.20, 0.82) | NC | NC |
| Cancer incidence (ICD-10 codes) |  |  |  |  |  |  |  |
| All malignant neoplasms (C00-C97) | 1.38  (1.08, 1.76) | 1.76  (1.38, 2.33) |  | 1.52  (1.11, 2.06) | 1.98  (1.52, 2.59) | 1.16  (0.77, 1.75) | 1.31  (0.60, 2.04) |
| Solid cancers (C00-C80) | 1.40  (1.09, 1.79) | 1.81  (1.39, 2.40) |  | 1.56  (1.14, 2.14) | 2.20  (1.52, 2.54) | 1.14  (0.75, 1.74) | 1.32  (0.59, 2.07) |
| Solid cancers other than thyroid | 1.19  (0.87, 1.64) | 1.71  (1.24, 2.37) |  | 1.50  (1.05, 2.14) | 2.18  (1.36, 2.54) | 0.60  (0.29, 1.26) | 0.78  (0.46, 1.99) |
| Hematopoietic cancers (C81-C96) | 0.73  (0.11, 5.01) | 0.39  (0.28, 5.40) |  | 0.09  (0.01, 0.68) | 0.34  (0.29, 2.29) | NC | NC |

HR, hazard ratio; CI, confidence interval; ICD-10, International Classification of Diseases and Related Health Problems, 10th Revision; NC, nonconvergence.

^a^Adjusted for employment status (binary), attained age (continuous), sex, birth year (<1960, 5-year intervals from 1960 to 1979, ≥1980), and years of employment duration (<1, 1-4, 5-9, ≥10).
